# Cutaneous Leishmaniasis, Sri Lanka

**DOI:** 10.3201/eid1307.060773

**Published:** 2007-07

**Authors:** Sujeevi S.K. Nawaratna, Danister J. Weilgama, Chandana J. Wijekoon, Manel Dissanayake, Kosala Rajapaksha

**Affiliations:** * University of Peradeniya, Peradeniya, Sri Lanka; † Teaching Hospital, Kurunegala, Sri Lanka; ‡ Teaching Hospital, Kandy, Sri Lanka; § Veterinary Research Institute, Peradeniya, Sri Lanka

**Keywords:** Leishmania donovani, cutaneous leishmaniasis, Sri Lanka, geographic distribution, clinical presentation, dispatch

## Abstract

Cutaneous leishmaniasis (CL) is an emerging disease in Sri Lanka. Of 116 patients with clinical symptoms suggestive of CL, 86 were confirmed positive for *Leishmania donovani*. Most patients had single dry lesions, usually on the face. Patients were from 5 of the 7 agroclimatic zones in Sri Lanka.

Leishmaniasis is a complex of diseases that has 3 main clinical forms, visceral, mucocutaneous, and cutaneous. For decades in Sri Lanka, leishmaniasis was considered an exotic disease. This changed in 1992 with the detection of locally acquired cases of cutaneous leishmaniasis (CL) (*1*,*2*). In 2003, Karunaweera et al. (*3*) reported that CL in Sri Lanka is caused by the parasite *Leishmania donovani* zymodeme MON-37; however, information on other aspects of the disease in Sri Lanka was scant. We present preliminary findings on the clinical manifestations and the distribution of CL in Sri Lanka.

## The Study

The study group consisted of patients referred by their dermatologists from June 2001 through June 2005 for skin lesions clinically suggestive of CL. The patients were examined for confirmatory diagnosis. Ethical clearance for the study was granted by the Research and Higher Degrees Committee of the Faculty of Medicine, University of Peradeniya, Sri Lanka.

Laboratory diagnosis was made by examination of Giemsa-stained touch or impression smears, in vitro culture, and/or by PCR. Tissue samples for cultures and molecular testing were obtained by use of a hypodermic needle. Evans modified Tobie medium was used for in vitro culture (*4*). PCR for diagnosis was performed on DNA extracted from tissue samples, by using a set of primers specific for all Old World *Leishmania* spp (*5*).

*Leishmania* DNA from 27 patients positive for CL and from 5 in vitro cultures (promastigotes) was sent to Laboratoire de Parasitologie, Besançon, France, for speciation. Characterization was performed by use of microsatellite analysis (also known as short tandem repeats), which used primers to amplify microsatellites in the internal transcribed spacer region of the *Leishmania* genomic DNA (*6*).

Of 120 patients examined, 4 had a history of travel overseas and were not included in the analysis. Of the remaining 116 patients, 86 (74.14%) were determined to be positive for CL. All but 2 of the 32 samples that were sent for speciation were identified as *L. donovani*; the 2 that were not identified did not have sufficient DNA for testing.

At the time of study, patients had CL-related lesions ranging in duration from 2 weeks to 4 years. Both male and female patients were infected (ages range 3–70 years).

All infected patients had cutaneous lesions only; none showed hepatosplenomegaly or enlargement of lymph nodes. Single lesions were seen in 70 of the 86 patients, and multiple lesions (range 2–5) were observed in the other 16 patients. Satellite lesions were seen in 11 of the 86 patients. Lesions appeared on the face of most patients (54.7%), but they also occurred in ears and on upper and lower limbs and the trunk (Table 1). No lesions were found from the waist to the knee.

**Table 1:** Distribution of cutaneous leishmaniasis lesions on infected patients, Sri Lanka, June 2001–June 2005

Location	No. patients*	%
Face	47	54.7
Scalp	1	1.2
Ear	5	5.8
Neck	3	3.5
Trunk	7	8.1
Upper limb		
Upper arm	4	4.7
Elbow	1	1.2
Forearm	22	25.6
Hand	2	2.3
Lower limb		
Above knee	0	0
Below knee	7	8.1
Foot	1	1.2

*Some had multiple lesions at different sites.

The lesions on most patients were dry and scaly (69/86), but on some, they were wet (17/86). A few patients showed a hypopigmented halo around the lesions. Patients’ lesions were categorized according to their appearance: of the 86 patients, 25 had papulonodular lesions, 25 had noduloulcerative lesions, and 36 had ulcerative lesions. Some ulcers had the typical volcanic appearance.

The CL-infected patients came from 12 administrative districts (Figure), representing 8 of the country’s 9 provinces. The areas in which the patients lived were representative of 5 of the 7 agroclimatic zones within Sri Lanka (Table 2).

**Figure:**
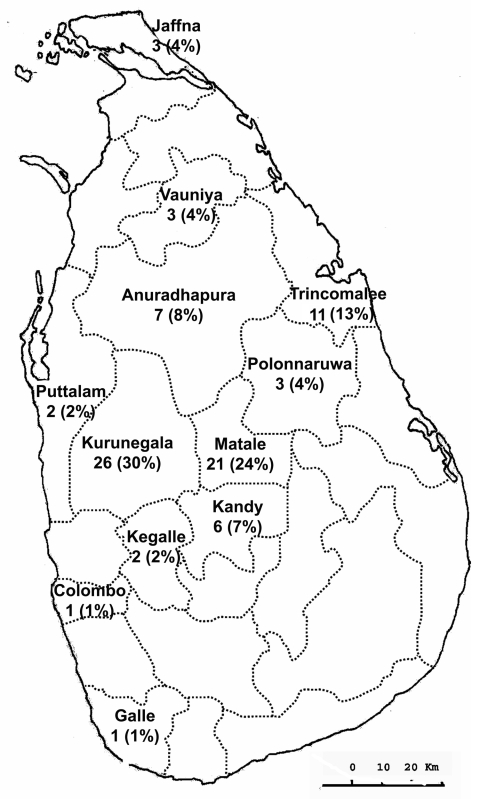
Geographic distribution of persons with cutaneous leishmaniasis in Sri Lanka during June 2001 through June 2005.

**Table 2:** Distribution of persons infected with cutaneous leishmaniasis in Sri Lanka, by agroclimatic zone, June 2001–June 2005

Agricultural zone (elevation), climatic zone (rainfall/year)	Patient’s district*	No. patients (N = 86)
Up country (>900 m)		
Wet (1,400 to >3,175 mm)	—	0
Intermediate (1,150 to >2,160 mm)	—	0
Mid country (300–900 m)		
Wet (1,270 to >3,175 mm)	Kandy	4
Intermediate (900 to >1400 mm)	Kandy	2
Low country (<300 m)		
Wet (1,525 to >2,540 mm)	Kegalle	2
	Colombo	1
	Galle	1
Intermediate (900 to >1,150 mm)	Kurunegala	26
Dry (<900 mm)	Puttalam	2
	Jaffna	3
	Anuradhapura	7
	Polonnaruwa	3
	Vauniya	3
	Trincomalee	11
	Matale	7

*The districts of Kandy and Matale fall into 2 agroclimatic zones.

## Conclusions

This study supports the earlier identification (*3*) of *L. donovani* as the causative agent of CL in Sri Lanka. The 27 isolates used in our study were from patients from 7 districts (Kandy, Matale, Kurunegala, Anuradhapura, Vauniya**,** Trincomalee, and Jaffna) in 5 Sri Lankan provinces (Central, North Central, North Western, Northern, and Eastern Provinces), representing different agroclimatic zones of the country. Thus, *L. donovani* appears to be the only species causing CL in Sri Lanka.

*L. donovani* usually causes visceral leishmaniasis, but there are other reports of it being associated with CL (*7*,*8*). A single parasite strain can cause different clinical symptoms (e.g., strains normally causing dermatotrophic symptoms instead causing visceral symptoms and vice versa) (*9*,*10*).

In our study, 68% of the infected persons were 11–40 years of age and frequently engaged in outdoor activities; similar findings have been reported from Guatemala (*11*). In addition, for patients in our study, lesions most often appeared on the face. Reports of CL from other countries and caused by different species have indicated similar findings (*8*,*12*). Lesions also frequently occurred on the forearms of our study population, which suggests that uncovered areas of the body are more prone to clinical manifestation of CL infection. Sharma et al. (*8*) also reported that lesions most commonly appeared on the face and then the upper limbs; an earlier report from Sri Lanka showed extensor surfaces of the limbs to be the most common site for lesions (*2*). We also found skin lesions on the chest and back of some adult males; however, lesions did not occur in these sites on females. This finding is a direct reflection of the cultural habits related to clothing in Sri Lankan society. Also of interest, none of the patients had lesions in the area from below the waist to above the knees. This finding again is due to clothing habits. Dedet et al. (*13*), in their study on CL in French Guiana, found the distribution of lesions on the body to depend on the form of dress. One patient in our study group had a lesion on the scalp. This appears to be an unusual site, as hairy parts of the body were otherwise unaffected.

The lesions in our patients were slow-progressing, and 58% appeared as papulonodular and noduloulcerative type lesions. A similar clinical picture was reported by Sharma et al. (*8*) in a study in which the researchers encountered noduloulcerative plaques with or without crusting. The morphologic appearance of lesions is known to vary depending on the species or strain of the causative organism (*13*) and the immune status of the patient (*14*).

Cutaneous nodules surrounded by areas of depigmentation have been described in atypical CL due to *L. chagasi* (*15*). This feature was also found in 12 of our 86 patients. Thus, the clinical manifestations of CL vary; there is not a characteristic clinical picture for disease caused by a particular species.

Most of the CL patients in our study were from the dry and intermediate zones of the low-altitude areas of Sri Lanka. No cases of CL were diagnosed in persons from high-altitude areas. This could be due to the abundance of sandflies (insect vectors for *Leishmania*) and their breeding sites in the low-altitude areas.

Since the first detection of CL in Sri Lanka in 1992, the number of cases detected annually has increased (*2*,*3*). A substantial number of persons in our study had a diagnosis of CL, but the prevalence of infection in Sri Lanka could not be determined because the study did not involve active case detection. To understand the atypical behavior of *L. donovani* in Sri Lanka, studies need to be directed toward understanding the vector bionomics and reservoir hosts for this parasite.
